# The association between serum activin A levels and albuminuria among community-dwelling middle-aged and older adults in Taiwan

**DOI:** 10.1038/s41598-021-99081-7

**Published:** 2021-10-08

**Authors:** Shih-Chen Chang, Chien-Yi Hsu, Li-Kuo Liu, Ya-Wen Lu, Yi-Lin Tsai, Ruey-Hsing Chou, Po-Hsun Huang, Liang-Kung Chen, Shing-Jong Lin

**Affiliations:** 1 Graduate Institute of Immunology, College of Medicine, National Taiwan University, Taipei, Taiwan; 2 Taipei Heart Institute, Taipei Medical University, Taipei, Taiwan; 3 Division of Cardiology, Department of Internal Medicine, School of Medicine, College of Medicine, Taipei Medical University, Taipei, Taiwan; 4 Division of Cardiology and Cardiovascular Research Center, Department of Internal Medicine, Taipei Medical University Hospital, No. 252, Wuxing St, Xinyi District, Taipei, Taiwan; 5 Division of Cardiology, Department of Medicine, Taipei Veterans General Hospital, Taipei, Taiwan; 6 Department of Critical Care Medicine, Taipei Veterans General Hospital, 112, No. 201, Sec. 2, Shih-Pai Road, Taipei, Taiwan; 7 Department of Medical Research, Taipei Veterans General Hospital, Taipei, Taiwan; 8 Center for Geriatrics and Gerontology, Taipei Veterans General Hospital, Taipei, Taiwan; 9 Institute of Clinical Medicine, National Yang Ming Chiao Tung University, Taipei, Taiwan; 10 Aging and Health Research Center, National Yang Ming Chiao Tung University, Taipei, Taiwan; 11 Cardiovascular Research Center, National Yang Ming Chiao Tung University, Taipei, Taiwan; 12 Taipei Municipal Gan-Dau Hospital, (managed by Taipei Veterans General Hospital), Taipei, Taiwan

**Keywords:** Biomarkers, Atherosclerosis, Kidney diseases

## Abstract

Activin A, a cytokine belonging to the transforming growth factor-β family, has been shown to play pivotal roles in tissue remodeling after renal injury and is present in elevated levels in diabetic patients. However, the association between activin A and albuminuria remains unclear. We aimed to evaluate their association by using cross-sectional data from community-dwelling middle-aged and older adults in Taiwan. We assessed 466 participants (67% male; mean age 71 ± 13 years) from the I-Lan Longitudinal Aging study for whom data pertaining to serum activin A level and urine albumin-to-creatinine ratio (UACR) were available. Of these, 323 (69%) had normal albuminuria, 123 (26%) had microalbuminuria, and 20 (4%) had overt albuminuria. Patients with overt albuminuria and microalbuminuria had significantly higher activin A concentrations than those in the normal albuminuria group (p < 0.001). Circulating activin A was significantly correlated with multiple risk factors, including higher systolic blood pressure and higher UACR. Univariate and multivariate results indicated that activin A level was an independent variable for albuminuria. The cutoff value of 602 pg/mL of activin A demonstrated a sensitivity of 70.6% and specificity of 75.7% (AUC 0.774) in diagnosing overt albuminuria. In conclusion, middle-aged and older adults with elevated activin A levels were associated with a higher incidence of albuminuria.

## Introduction

Albuminuria, a major presentation of chronic kidney disease caused by the deterioration of glomerular filtration function and increased excretion of albumin, has become a worldwide public health issue^1,2^. Although it is a known predictor of poor renal function and is associated with all-cause mortality, the underlying mechanism of inflammatory cytokines in albumin secretion remains unclear^3,4^. Clinically, traditional renal protection strategies, such as systolic pressure control and inflammation control, have been employed^5,6^. However, recent studies have provided evidence that glomerular integrity gets irreversibly disrupted even before the development of albuminuria. As albuminuria a risk factor, prognostic marker, and therapeutic target for renal disease progression, it is essential to discover novel surrogate markers and potential therapeutic targets to treat this condition^7,8^. The worsening of albuminuria is associated with an increased risk of renal and cardiovascular disease progression^9^. In addition to the complicated clinical scenario of both acute and chronic renal injury, inflammatory cytokines, coordinated by innate cells and glomerular cells, including podocytes, tubular cells, and endothelial cells, play an important role in causing albuminuria and renal function deterioration^10,11^. Furthermore, albuminuria and the progression of renal disease are interwoven and mutually determined. Increasing evidence has indicated that pro-inflammatory cytokine-mediated tubulointerstitial damage is the predominant cause of renal disease progression^12^. Although multiple factors, including Interleukin-1, Interleukin-6, and transforming growth factor-β (TGF-β), have been reported to play roles in renal fibrosis, little is known about the association and role of activin A in albuminuria.

Activin A, a member of the TGF-β superfamily, has multifaceted roles in regulating diverse cellular processes, including inflammation, cell death, facilitating endocrine function, and even human T_FH_ cell differentiation^13–15^. It regulates physiological and pathological events through the activin type II receptor, associated with the Smad proteins (Smad2 and Smad3)-dependent pathway. Recently, activin A with pro-inflammation and pro-fibrotic properties has been reported to play pivotal roles in chronic inflammation and tissue fibrosis, as seen in an animal model-based study where it was shown that it played an important role in the development of interstitial renal fibrosis, leading to albuminuria. The association between part of the spectrum of chronic diseases and activin-A has already hinted by previous study. In our recent study, an elevated circulating activin A level was associated with elevated systolic blood pressure and pulse pressure^19^. Additionally, it was also associated with pre-diabetic and diabetic patients. As such, we concluded that the activin A level was an independent risk factor for pre-diabetes and diabetes^20^. In pre-diabetic patients, its level was positively correlated with the carotid intima-media thickness.

Internationally, the proportion of people over 65 in the total population is 7%, 14%, and 20%, which are called aging society, aged society, and super-aging society, respectively. Taiwan has become an aging society in 1993, and has achieved aged society since 2018. It is estimated that it will enter a super-aged society in 2025^35^. In 2020, the super-old (over 85 years old) population accounted for 10.7% of the elderly population, and it will increase to 27.4% in 2070. Among all countries, Taiwan has the highest incidence rate of end-stage renal disease (ESRD), hence studies for the clinically recognized chronic kidney disease (CKD) has been yielded. Kuo et al. disclosed elevation of clinically recognized CKD from 1.99% in 1996 to 9.83% in 2003^36^. Also, under multivariate model, with an odds ratio of 13.95 being calculated for the group with age greater than 75-year-old in comparison to the group 20 years younger. The epidemiology data in Taiwan also concluded older age as a risk factor for CKD, which may be directly or indirectly associated with comorbidity developed with aging process^37^.

The link that activin A has a systemic role in chronic diseases throughout multiple systems was yet established with early onset disease status and aging process. However, direct evidence on the association between circulating activin A and albuminuria is lacking. To our interest, activin A could serve as a biomarker for albuminuria and for identifying the disease stage. Collectively, we tested the hypothesis that elevated circulating activin A concentration is associated with microalbuminuria and overt albuminuria.

## Results

### Serum activin A level is associated with the extent of albuminuria

Inflammaging has been reported to be the dominant physiological change in older population^16^. The role of activin A in the cytokine spectrum of inflammation and kidney pathophysiology remains unclear. Thus, in our study, patients’ basic demographic, clinical characteristics, and serum activin A levels were surveyed (Table 1). A total of 466 participants from the ILAS study were eligible for this study. Among them, 323 (69.3%) had normal albuminuria, 123 (26%) had microalbuminuria, and 20 (4%) had overt albuminuria (Table 1). Participants with microalbuminuria and overt albuminuria were older and more likely to be diagnosed with diabetes mellitus, high systolic blood pressure (SBP), higher body mass index, higher urine albumin to creatinine ratios (UACRs), higher serum triglyceride levels, higher uric acid levels, higher HbA1c, and higher activin A levels. However, they had significantly lower total and high-density lipoprotein cholesterol levels and glomerular filtration rates (GFRs) than subjects with normal albuminuria (all p < 0.01). Similar to previous studies, most of these factors, including systolic blood pressure and renal function, are highly associated with human aging. Interestingly, activin A, an age-associated parameter in our study, was specifically associated with the different severities of albuminuria.

**Table 1:** Comparison of general characteristics among the different albuminuria status.

	Normal albuminuria (n = 323)	Microalbuminuria (n = 123)	Overt albuminuria (n = 20)	*p* value	*p trend*
Age, years	67.6 ± 8.8	71.9 ± 8.7	73.5 ± 11.1	< 0.001**	0.004*
Sex, men	161 (50)	61 (50)	11 (55)	0.900	0.656
BMI	24.4 ± 3.2	25.3 ± 4.3	26.2 ± 4.4	0.009*	0.031*
Waist circumference, cm	85.3 ± 9.1	87.5 ± 10.2	90.0 ± 11.1	0.020*	0.036*
Appendicular skeletal muscle mass, kg	17.7 ± 4.1	17.5 ± 3.5	18.1 ± 3.8	0.746	0.650
Mini-Nutrition Assessment	27.0 ± 2.0	26.9 ± 2.0	26.7 ± 2.2	0.808	0.556
Current smoking	61 (19)	33 (27)	1 (5)	0.039*	0.134
Hypertension	148 (46)	70 (57)	16 (80)	0.003*	0.003*
Diabetes	49 (15)	32 (26)	7 (35)	0.006*	0.027*
CAD	11 (3)	5 (4)	3 (15)	0.039*	0.011*
SBP, mmHg	131.4 ± 15.0	142.2 ± 18.7	145.9 ± 23.4	< 0.001**	< 0.001**
Fasting glucose, mg/dL	99.6 ± 25.3	107.5 ± 36.2	120.9 ± 51.3	0.001*	0.003*
eGFR, ml/min	75.2 ± 22.7	69.1 ± 26.8	56.1 ± 27.0	< 0.001**	0.001*
ALT, U/L	28.1 ± 22.6	28.6 ± 20.5	28.6 ± 11.3	0.973	0.927
Total cholesterol, mg/dL	193.7 ± 33.4	190.4 ± 38.3	187.6 ± 38.5	0.536	0.448
HDL-C, mg/dL	54.0 ± 13.4	51.1 ± 11.7	48.5 ± 12.9	0.029*	0.064
LDL-C, mg/dL	120.9 ± 33.7	119.3 ± 36.5	110.3 ± 40.1	0.398	0.183
Triglyceride, mg/dL	122.7 ± 96.3	133.3 ± 108.6	215.7 ± 419.9	0.008*	0.002*
Uric acid, mg/dL	5.9 ± 1.4	5.9 ± 1.5	6.4 ± 1.3	0.346	0.146
Homocystein, μmol/L	13.9 ± 7.1	15.2 ± 5.8	16.2 ± 5.4	0.071	0.134
hs-CRP, mg/L	1.9 ± 3.5	2.8 ± 4.3	2.4 ± 3.4	0.064	0.523
Activin A, pg/mL	503.6 ± 160.5	579.9 ± 193.2	665.9 ± 151.1	< 0.001**	< 0.001**
Follistatin, pg/mL	1587 ± 1372.5	1699.8 ± 620.3	2116.9 ± 990.8	0.133	0.057
IGF-1, ng/mL	123.1 ± 56.0	117.2 ± 43.1	108.1 ± 36.2	0.316	0.225
HOMA-IR,unit	1.9 ± 1.6	2.5 ± 2.8	9.5 ± 25.3	< 0.001**	< 0.001**
UACR, mg/g	8.4 ± 4.6	52.8 ± 39.1	914.9 ± 660.9	< 0.001**	< 0.001**

*p < 0.05, **p < 0.001.

Values are mean ± SD or n (%); *ALT* aminotransferase, *BMI* body mass index, *CAD* coronary artery disease, *eGFR* estimated glomerular filtration rate, *hs-CRP* high-sensitivity C-reactive protein, *HDL-C* high-density lipoprotein cholesterol, *HOMA-IR* homeostasis model assessment-estimated insulin resistance, *IGF-1* insulin-like growth factor-1, *LDL-C* low-density lipoprotein cholesterol, *SBP* systolic blood pressure, *UACR* urine albumin-to-creatinine ratio.

**Figure 1:**
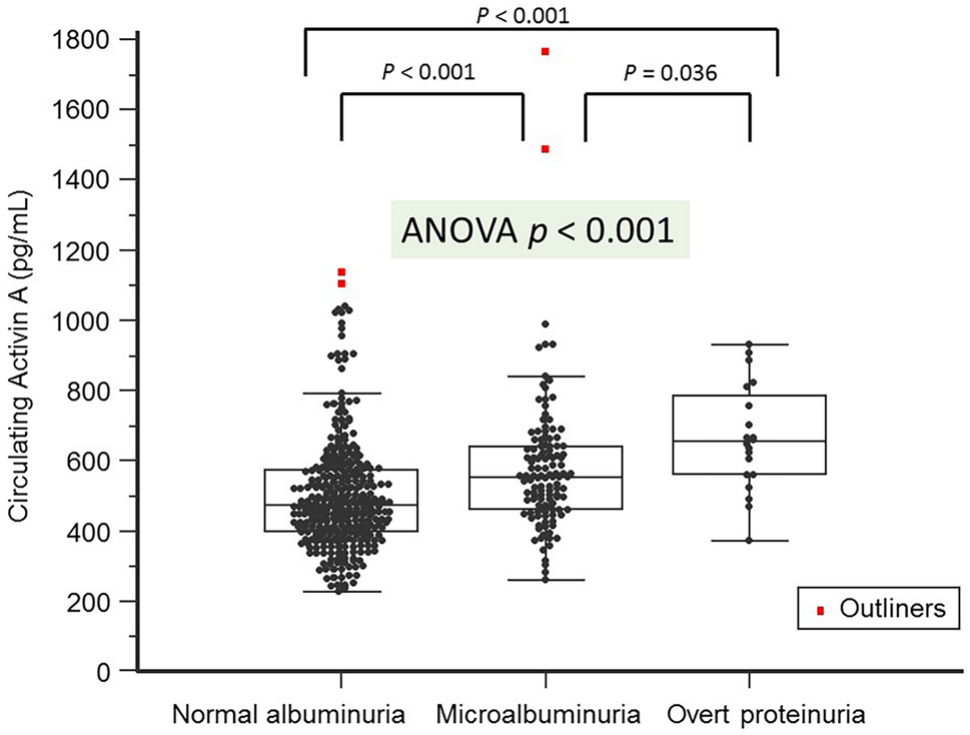
Serum activin A elevation is positively correlated to the severity of albuminuria.

**Figure 2:**
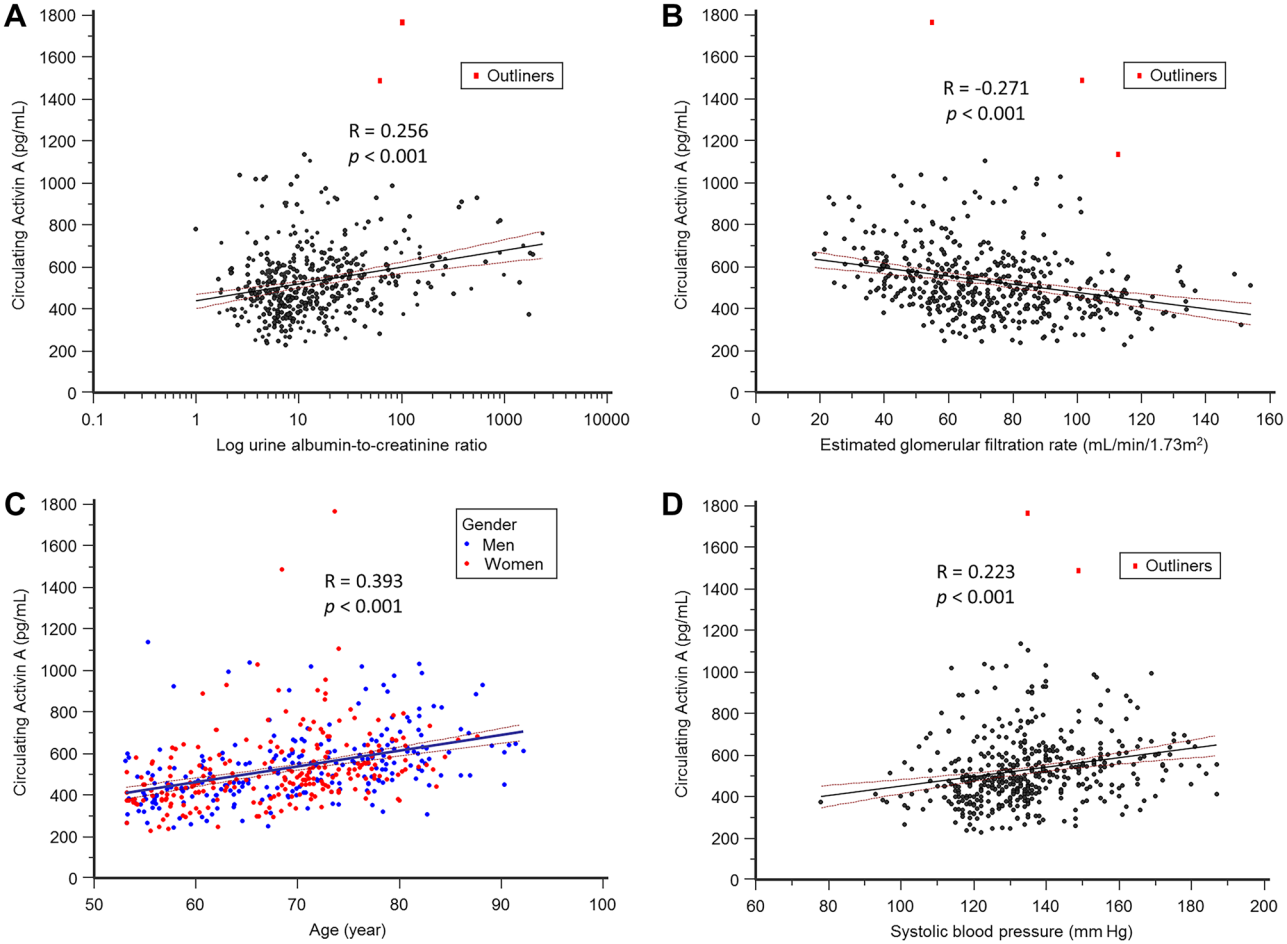
The elevations in circulating activin A levels are correlated to the aging-related demographic parameters.

As seen in Fig. 1, subsequent analysis using ANOVA further justified that the significant increase (p < 0.001) in serum activin A level was associated with the elevated extent of albuminuria. This finding supports our hypothesis that increased circulating activin A level may directly or indirectly alter the integrity of glomerular function, causing albumin leakage. As shown in Fig. 2, SBP, eGFR, logUACR, and age were also significantly correlated with elevated activin A levels (p < 0.001).

### Statistical evidence showed that elevated activin A was independently associated with albuminuria

Although hypertension plays a significant role in the development of albuminuria, the activin A-driven disturbance of glomerular function has been found by a previous study to play an equally significant role^17^. In Table 2, we utilized logUACR, a more sophisticated parameter, to normalize albuminuria, and the univariate and multivariate analyses results indicated an association between elevated activin A and logUACR. The results presented in Table 2 further confirm the prior findings (Fig. 1 and Table 1) that elevated circulating activin A was positively associated with albuminuria. Table 3 summarizes the results of the univariate and multivariate logistic regression analyses, which showed that SBP (p < 0.001 in both analyses) and activin A levels were significantly associated with albuminuria (p < 0.001 in the univariate analysis, p < 0.012 in the multivariate analysis).

**Table 2:** Univariable and multivariable associations with log urine albumin-to-creatinine ratio (LogUACR).

	Univariate analysis		Multivariate analysis^†^	
	Coefficient	*p* value	Coefficient	*p* value
Age, years	0.248	< 0.001**	0.028	0.686
Sex, men	0.048	0.306		
Body mass index	0.113	0.015*	0.125	0.070
Waist circumference, cm	0.096	0.015*	− 0.093	0.173
Current smoking	0.023	0.620		
Use of antihypertensive drug	0.115	0.013*	0.038	0.413
Systolic blood pressure, mmHg	0.328	< 0.001**	0.273	< 0.001**
Fasting plasma glucose, mg/dL	0.170	< 0.001**	0.106	0.031*
Estimated glomerular filtration rate, mL/min/1.73 m^2^	− 0.224	< 0.001**	− 0.198	0.008*
HDL-cholesterol, mg/dL	− 0.065	0.162		
Triglycerides, mg/dL	0.103	0.026*	− 0.019	0.757
Homocysteine, μmol/L	0.117	0.012*	− 0.017	0.732
High-sensitivity C-reactive protein, mg/L	0.032	0.508		
Activin A, pg/mL	0.256	< 0.001**	0.122	0.012*
Follistatin, pg/mL	0.064	0.169		
HOMA-IR, unit	0.166	< 0.001**	0.121	0.066

*p < 0.05, **p < 0.001.

^†^The multivariate regression model included all available variables with a p-value < 0.100.

*eGFR* estimated glomerular filtration rate, *hs-CRP* high-sensitivity C-reactive protein.

**Table 3:** Logistic regression analysis of risk factors associated with microalbuminuria and overt albuminuria.

	Univariate		Multivariate	
	Odds ratio (95% CI)	p value	Odds ratio (95% CI)	p value
Age, 1 SD = 9.1 year	1.66 (1.35–2.01)	< 0.001**	1.34 (1.04–1.72)	0.022*
Sex, men	1.03 (0.69–1.52)	0.895		
Body mass index, 1 SD = 3.6	1.33 (1.09–1.63)	0.005*	1.26 (0.87–1.81)	0.221
Waist circumference, 1 SD = 9.5 cm	1.30 (1.06–1.58)	0.010*	0.85 (0.60–1.22)	0.375
Current smoking (yes or no)	1.34 (0.83–2.16)	0.228		
Use of antihypertensive drug (yes or no)	1.57 (1.03–2.41)	0.037*	1.25 (0.74–2.10)	0.404
Systolic blood pressure, 1 SD = 17.2 mmHg	1.99 (1.61–2.48)	< 0.001**	1.93 (1.50–2.48)	< 0.001**
Fasting plasma glucose, 1 SD = 30.5 mg/dL	1.36 (1.10–1.68)	0.004*	1.37 (1.10–1.70)	0.005*
Estimated glomerular filtration rate, 1 SD = 24.5 mL/min/1.73 m^2^	0.71 (0.58–0.88)	0.001*	0.83 (0.55–1.25)	0.372
HDL-cholesterol, 1 SD = 13.0 mg/dL	0.76 (0.62–0.94)	0.012*	0.77 (0.60–0.98)	0.034*
Triglycerides, 1 SD = 131.2 mg/dL	1.17 (0.96–1.44)	0.126		
Homocystein, 1 SD = 6.8 μmol/L	1.25 (1.01–1.55)	0.041*	0.96 (0.75–1.24)	0.766
High-sensitivity CRP, 1 SD = 3.7 mg/L	1.25 (1.02–1.52)	0.031*	1.15 (0.93–1.43)	0.207
Activin A, 1 SD = 173.9 pg/mL	1.66 (1.35–2.06)	< 0.001**	1.32 (1.05–1.67)	0.017*
Follistatin, 1 SD = 1201.2 pg/mL	1.15 (0.91–1.46)	0.240		
HOMA-IR, 1 SD = 1.9 unit	1.33 (1.11–1.59)	0.002*	1.08 (0.90–1.30)	0.428

*p < 0.05, **p < 0.001.

*CRP* C-reactive protein, *HDL* high-density lipoprotein, *HOMA-IR* homeostasis model assessment-estimated insulin resistance.

### Serum activin A level is a positive predictor of albuminuria

**Figure 3:**
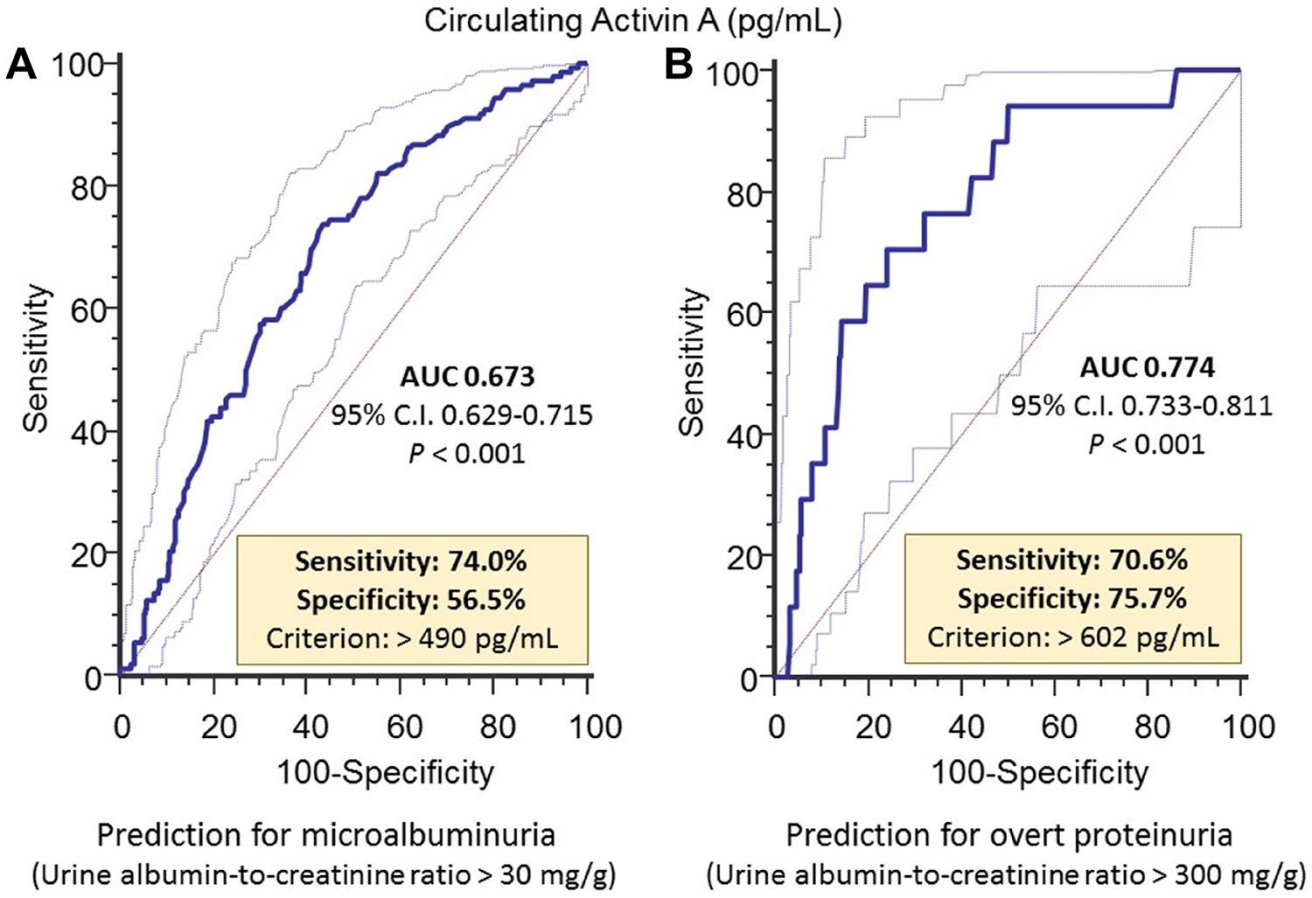
A receiver operating characteristic curve analysis between circulating activin A level and (**A**) microalbuminuria (urine albumin-to-creatinine ratio > 30 mg/g), (**B**) overt albuminuria (urine albumin-to-creatinine ratio > 300 mg/g).

Subsequently, we tested whether activin A could be a feasible and accurate predictor of albuminuria in clinical practice through a receiver operating characteristic (ROC) analysis (Fig. 3). Using the maximal Youden index, the optimal cutoff point of activin A level in the albuminuria group was determined to be at 490 pg/mL (sensitivity 74%, specificity 56.5%), with an area under the curve (AUC) of 0.673. Using the similar method, the optimal cutoff point in the overt albuminuria group was set at 602 pg/mL (sensitivity, 70.6%; specificity, 75.7%), with an AUC of 0.774.

## Discussion

The prevalence of chronic kidney disease has been increasing due to the increasing age of the population^38^. Thus, a comprehensive and predictive marker is required. To date, the association between glomerular function integrity and albuminuria has been well established. However, the deterioration of albuminuria was considered under the progressive loss of renal function, caused by a bundle of mechanisms either in a string or in parallel^18,19^. Microalbuminuria, ranging from 30 to 300 mg/day of urine albumin, is prevalent in patients with hypertension, diabetes, and renal disease. It is also present in healthy individuals. Our study tested the association, sensitivity, and specificity of circulating activin A with the various severities of albuminuria. Consistently, in a murine model, the elevation of activin A was associated with both aging and biomechanical cardiac stress. Activin A activation through the activin type II receptor induces the ubiquitin pathway, leading to the degradation of sarcoplasmic reticulum Ca^2+^ ATPase and loss of myocyte function^20^. Our data show that activin A could be an early indicator for the prescription of cardiorenal-protective regimens. To the best of our knowledge, this study was the first to reveal an association between serum activin A and albuminuria. Interestingly, we found that increased activin A levels were associated with decreased GFRs and elevated albuminuria levels. Moreover, the multivariate analysis indicated that, similar to hypertension, which is a significant cause of albuminuria, increased activin A levels were also linked to micro- and macroalbuminuria.

In this study, we demonstrated that elevated activin A level was an independent risk factor for albuminuria. In our previous study, elevated activin A was associated with SBP and pulse pressure^21^. To date, a basic study has proven that activin A can regulate blood pressure through the renin–angiotensin system^22^. Therefore, we could infer from the previous study’s findings that activin A could cause albuminuria by up-regulating the SBP and pulse pressure, though there is lacking direct evidence on its underlying mechanism. Altogether, this study revealed the significance of serum activin A, which is directly or indirectly associated with albuminuria.

Our recent study also reported that elevated activin A was associated with pre-diabetic and diabetic patients^23^. The progression of diabetes may be driven by a chronic onset systemic disease and predisposing factors, including altered metabolic and chronic inflammation. Diabetic retinopathy and diabetic renal disease, proven to be involved in vessel remodeling and endothelial cell injury, are the two most common diseases that manifest early in diabetic patients. Glucose itself is not the only factor leading to tissue remodeling as other inflammatory factors, such as the TGF-β superfamily, may also be implicated^17^. Thus, in a previous study, we provided evidence that elevated serum activin A levels were associated with carotid intima-media thickness, showing that activin A was a crucial predisposing factor in the disruption vessel morphology integrity among diabetic patients. This idea and conclusion could be extended to this study since elevated activin A was significantly associated with albuminuria. Based on the evidence that activin A disrupts vessel morphology, we proposed that the renal artery and afferent arterioles were also affected to a certain extent, accompanied by endothelial cell dysfunction, resulting in albuminuria in patients with elevated activin A levels. Although whether activin A alters the renal vascular system's homeostasis and the underlying mechanism governing association between serum activin A levels and albuminuria remain unclear, we concluded that systemic elevated activin A should be a positive associated risk factor for albuminuria.

In this study, the cutoff point of 490 pg/mL for the circulating activin A in the albuminuria group yielded a sensitivity of 74% and a specificity of 56.5%. Meanwhile, the cutoff point of 602 pg/mL in the overt albuminuria group lead to a sensitivity of 70.6% and specificity of 75.7%. The causal mechanism of activin A-driven albuminuria is complicated. The structural and functional integrity of the trinity of the glomerular infiltration structure, including endothelial cells, podocytes, and glomerular basement membranes, is directly associated with albuminuria. In this study, we dissected the link between activin A and the integrity of the glomerular infiltration structure layer by layer. First, as mentioned in previous studies, albuminuria is associated with systemic endothelial cell dysfunction. A recent cross-sectional, population-based study reported an association between albuminuria and reduced capillary density through skin capillaroscopy^24^. Moreover, a recent study indicated a positive association between activin A and hypertension and suggested that in the scenario of a chronic disease progression, endothelial cell dysfunction was caused by multiple mechanisms. The lining of endothelial cells in the glomerular infiltration structure limits the passage of albumin. Thus, any interruptions in this delicate lining may amplify the level of albuminuria. Activin A has also been reported to induce endothelial cell dysfunction, contributing to the imbalance of endothelial-podocyte crosstalk, a major cause of albuminuria. Second, although activin A is essential for the differentiation of human stem cells into podocytes, a chronically elevated level of the pro-inflammatory type potentiates fibroblast activation, replacing injured cells with fibrosis deposition^25,26^. Immunological stresses driven by circulating pro-inflammatory cytokines are the predominant causes of podocyte and endothelial cell dysfunction. Activin A, which has pro-inflammatory and pro-fibrotic properties, has also been found to play pivotal roles in chronic inflammation and tissue fibrosis, such as pulmonary, liver, and interstitial renal fibrosis. As a member of the TGF-β superfamily, activin A shares similar properties with TGF-β, which plays a substantial role in glomerular sclerosis and podocyte dysfunction^27–30^ through the activation of the TGF-β receptors on podocytes and endothelial cells. As such, cytokines related to the TGF-β superfamily may also cause podocyte and endothelial cell dysfunction. However, no human study has discovered the relationship between activin A levels and albuminuria. Although our study highlights that activin A could have a role in interrupting the integrity of glomerular filtration, leading to albuminuria, further translational studies are needed to establish its mechanism.

In the progression of renal function loss, acute renal injury, chronic inflammation, and tissue repair affect the overall outcome. As observed in a senescence study^31^, TGF-β has been reported to trigger senescence cells to express p21 and p16, leading to cell cycle arrest. As a member of the TGF-β superfamily, activin A has also been reported to be part of the senescence secretome^32^. Systemically elevated activin A may contribute to the senescence of renal tubular epithelial cells. Post-cell injury Senescence is a natural mechanism for immune surveillance and prevention of tumor cell generation. During phase senescence, cells implement a complicated pro-inflammatory response, termed the senescence-associated secretory phenotype (SASP)^32^. The secretome of senescence is the most important signal in the activation of immune responses. In a recent study, activin A was also reported to play a role in paracrine senescence. On the other hand, in acute kidney injury, a recent study indicated that activin A, which is primarily secreted by the proximal tubular cell, is associated with tissue injury and tissue repair inhibition. Tubular cell injury was expected to impair the endocytic filtration of albumin, resulting in the worsening of albuminuria. In summary, our study showed that activin A elevation linked to albuminuria might be a chronic outcome driven by renal tubular epithelial senescence^3,32–34^.

Though the mechanism by which activin A drives albuminuria remains unclear, tissue injury and inflammation mechanisms may play crucial roles in this process. To the best of our knowledge, the present study is the first to disclose the association between albuminuria and serum activin A levels. In this study, we found that increased activin A levels were related to albuminuria, similar to hypertension.

In conclusion, middle-aged and older adults with elevated activin A levels were associated with a higher incidence of microalbuminuria/overt albuminuria, independent of hypertension or diabetes. Circulating activin A levels might be a positive associated factor of albuminuria. Further studies are warranted to assess the causal relationships and clinical relevance.

## Material and methods

### Study population

The I-Lan Longitudinal Aging study (ILAS) is a community-based cohort study for the middle-aged and older adults in I-Lan County of Taiwan. This study was approved by the institutional review board of the National Yang Ming University (approval no. YM103008). The ILAS study aimed to evaluate the complex associations between age and multiple factors, including hormones, comorbidities, sarcopenia, and cognitive function. A random selection of community-dwelling adults above 50 years of age in I-Lan County, Taiwan was performed in this study. The exclusion criteria were: (i) inhabitants who were unable to communicate with the research nurse or grant an interview; (ii) inhabitants who were unable to complete the evaluation tests due to poor functional status; (iii) inhabitants who had limited life expectancy (< 6 months) due to major illnesses; and (iv) current residents of long-term care facilities. We conducted our study in compliance with the recognized international standards, including the principles of the Declaration of Helsinki. All participants had given their written informed consents, and the study was approved by the Institutional Review Board (IRB) of the National Yang-Ming University, Taipei, Taiwan (IRB: YM103008).

### Demography, physical examination, and laboratory examination

The patients’ medical history including events of chronic renal diseases, cardiovascular diseases, and cerebrovascular diseases, and risk factors were documented through a personal interview. Weight, height, and waist circumference were measured, and the body mass index was calculated. Brachial blood pressure was assessed with a sphygmomanometer after patients had been sitting for more than 15 min. The average of three SBP measurements was used for analysis in this study. All blood samples were drawn with the participant in a seated position after a 10-h overnight fast. Serum activin A levels were measured using an enzyme-linked immunosorbent assay. Serum concentrations of glucose, total cholesterol, triglyceride, low-density lipoprotein cholesterol, and high-density lipoprotein cholesterol were determined using an automatic analyzer (ADVIA 1800, Siemens, Malvern, PA, USA). Whole-blood glycated hemoglobin A1c was measured by an enzymatic method using a Tosoh G8 HPLC Analyzer (Tosoh Bioscience, San Francisco, CA, USA). The serum levels of hs-CRP, homocysteine, and insulin-like growth factor-1 were also measured.

### Measurement for urinary albumin excretion and definition of albuminuria

A single voided morning urine sample was used to measure the UACR (mg/g). UACR measured in a spot urine sample is highly correlated with 24-h urine albumin excretion. The presence of microalbuminuria is defined as a UACR of 30–300 mg/g. The presence of overt albuminuria is defined as a UACR > 300 mg/g.

### Statistical analysis

The analysis was performed on the complete data set, and the results are expressed as mean ± SD or percentage frequency. Analysis of variance (ANOVA) was used to compare the continuous variables among the three groups. Logistic regression analysis was performed to evaluate the association between the activin A levels and the various factors. A univariate and multivariate approach was used to analyze the association between the activin A levels and associated parameters. ROC analysis was also performed to evaluate the predictive accuracy of activin A in the diagnosis of microalbuminuria and overt albuminuria. Data were analyzed using the SPSS software (version 20, SPSS, Chicago, IL, USA). A p-value of < 0.05 was considered to indicate statistical significance.
